# The history of olive cultivation in the southern Levant

**DOI:** 10.3389/fpls.2023.1131557

**Published:** 2023-02-23

**Authors:** Oz Barazani, Arnon Dag, Zachary Dunseth

**Affiliations:** ^1^ Agricultural Research Organization, Institute of Plant Sciences, Department of Vegetables and Field Crops, Rishon LeZion, Israel; ^2^ Agricultural Research Organization, Institute of Plant Sciences, Department of Fruit Tree Sciences, Gilat Research Center, Gilat, Israel; ^3^ Joukowsky Institute for Archaeology and the Ancient World, Brown University, Providence, RI, United States

**Keywords:** archaeobotany, crop wild relatives, landraces, olives, palynology

## Abstract

The olive tree (*Olea europaea* L. subsp. *europaea* var. *europaea*) is one of the most important crops across the Mediterranean, particularly the southern Levant. Its regional economic importance dates at least to the Early Bronze Age (~3600 BCE) and its cultivation contributed significantly to the culture and heritage of ancient civilizations in the region. In the southern Levant, pollen, pits and wood remains of wild olives (*O. europaea* subsp. *europaea* var. *sylvestris*) has been found in Middle Pleistocene sediments dating to approximately 780 kya, and are present in numerous palynological sequences throughout the Pleistocene and into the Holocene. Archeological evidence indicates the olive oil production from at least the Pottery Neolithic to Chalcolithic transition (~7600-7000 BP), and clear evidence for cultivation by, 7000 BP. It is hypothesized that olive cultivation began through the selection of local genotypes of the wild var. *sylvestris*. Local populations of naturally growing trees today have thus been considered wild relatives of the olive. However, millennia of cultivation raises questions about whether genuine populations of var. sylvestris remain in the region. Ancient olive landraces might thus represent an ancient genetic stock closer to the ancestor gene pool. This review summarizes the evidence supporting the theory that olives were first cultivated in the southern Levant and reviews our genetic work characterizing local ancient cultivars. The significance and importance of old cultivars and wild populations are discussed, given the immediate need to adapt agricultural practices and crops to environmental degradation and global climate change.

## 1 Introduction

The cultivated olive tree (*Olea europaea* L. subsp. *europaea* var. *europaea*) is among the most iconic and important crops native to the Mediterranean Basin. By the fifth millennium BCE, cultivation of olives was widespread in the region (Zohary et al., 2012) and had altered the agricultural landscape of the southern Levant (an area covering modern-day Syria, Lebanon, Israel, the Palestinian Authority, and western Jordan). Archaeological evidence suggests that by the Early Bronze Age (4^th^-3^rd^ millennium BCE) trade in table olives and olive oil was extensive (Zohary et al., 2012; Langgut et al., 2019), and Late Bronze Age shipwrecks and texts (such as the 14^th^ century Ugaritic tablets), show the international character of olive trade by the late second millennium BCE. The olive tree is mentioned numerous times in the Hebrew and Christian bibles, as well as the Quran, demonstrating the importance of the tree to the cultural heritage of people in the southern Levant (Kaniewski et al., 2012). Olives and olive cultivation are mentioned extensively in ancient Roman agricultural texts, including those by Cato (*De Agricultura*, second century BCE) and Columella (*De re rustica*, first century CE), as well as in the Hebrew Mishna and Talmud (third century CE). Beyond a daily provision, the biblical text suggests ancient Israelites used olive oil in the ordination of high priests and kings (Exodus 30:33) and as fuel for lamps (Exodus 27:20, Leviticus 24:2, see also Welch, 2022). The olive also symbolized peace and prosperity in the bible (Genesis 8:11, Deuteronomy 8:8), and victory and wisdom in ancient Greece (Therios, 2009).

Several important agricultural crops were first domesticated and cultivated in the eastern Mediterranean, including wheat, barley, lentil, chickpea, pea, flax, and olive (Zohary et al., 2012). The cultivation and domestication of annual grains such as wheat and barley preceded those of fruit trees, which required a different knowledge of vegetative propagation (Zohary and Spiegel-Roy, 1975; Spiegel-Roy, 1986; Larson et al., 2014; Abbo et al., 2015). Crop wild relative (CWR) populations of many species are scattered around the southern Levant, including naturally growing olive trees. These trees, *O. europaea* subsp. *europaea* var. *sylvestris* (Mill) Lehr, are thought to be the ancestor of cultivated olives (Zohary et al., 2012) and are considered ‘genetic founder stocks’ for cultivated olives (Lev-Yadun et al., 2000). Olive landraces, which presumably encompass a higher level of genetic diversity than their modern decedents (cf. Zhang et al., 2017), might thus represent a genetic stock closer to the ancestor gene pool. However, the long history of olive cultivation and likely hybridizations between wild and cultivated variants raises the question whether genuine populations of var. *sylvestris* still exist (Barazani et al., 2016).

It is generally accepted that the cultivation of olive trees started through selection from natural populations of wild *O. europaea* subsp. *europaea* var. *sylvestris* (Mill) Lehr (Zohary and Spiegel-Roy, 1975; Terral et al., 2004; Kaniewski et al., 2012). It is still unclear whether the domestication of crops was initiated by the conscious selection of desirable traits (e.g., Abbo et al., 2012; Spengler, 2020). Nevertheless, it is reasonable to assume that the cultivation of olives started through the propagation of ‘better’ phenotypes, e.g., trees with larger fruits, high oil content, increased yield, etc. (Zohary and Spiegel-Roy, 1975; Zohary et al., 2012). Additionally, it is broadly accepted that the cultivation of fruit crops in general—and olive specifically—was strongly associated with the invention of grafting (Zohary et al., 2012). However, the ability for vegetative cultivation of olives by removing and transplanting truncheons suggests that olive cultivation might have begun independently from the invention of grafting (Foxhall, 2007).

Several recent publications have summarized the domestication process of the olive tree (Kaniewski et al., 2012; Besnard et al., 2013b; Diez et al., 2015; Besnard and Rubio de Casas, 2016; Díez and Gaut, 2016; Besnard et al., 2018; Langgut et al., 2019). Many aspects of domestication have been debated, including where domestication first occurred, and whether there were multiple domestication events. Besnard et al. (2013b) suggested that the domestication of olive trees started in the northern Levant and was followed by secondary diversification around the Mediterranean Basin. More recently, an alternative scenario describing two independent centers of domestication in the eastern and central parts of the Mediterranean area has been postulated (Diez et al., 2015). The contradicting theories of single vs. multiple independent domestication centers of olive have reinvigorated the debate regarding the origins and processes of olive domestication and cultivation (Besnard and Rubio de Casas, 2016; Díez and Gaut, 2016). A third hypothesis recently put forth by Langgut et al. (2019), based on the palynological record, suggests that the southern Levant was the center of initial cultivation from which other domestication centers evolved. Based on this hypothesis, this review summarizes the history of olive cultivation and usage in southern Levant, from the Chalcolithic through the Bronze and Iron Ages, and emphasizes the potential and importance of combining different scientific disciplines when studying crop domestication in general, and olives specifically. It also summarizes the southern Levantine genetic evidence emphasizing the value of local plant genetic resources, including both the wild olive relative and local landraces.

## 2 The beginning of olive cultivation in the southern Levant as reflected by different scientific disciplines

### 2.1 Palynological evidence

Olives produce large amounts of pollen, typical of wind-pollinated species (Cuevas and Polito, 2004). Consequently, olive pollen has been found in various geological layers in the southeast Mediterranean (we herein refer primarily to evidence from Israel and the Palestinian Authority), including outside its cultivated zone and the natural distribution range of the wild var. *sylvestris* (e.g., the Dead Sea region). Pollen is considered a reliable bio-marker that assists in constructing past climate and vegetation structures (Schiebel, 2013; Finkelstein and Langgut, 2018). Evidence of ancient pollen, as well as pits and wood remains from the site of Gesher Benot Ya’akov (approximately 780 kya BP) indicates that wild olives (var. *sylvestris*) existed in the region as early as the Middle Pleistocene (Goren-Inbar et al., 2000; Goren-Inbar et al., 2002; Van Zeist and Bottema, 2009; Melamed et al., 2016; Goren-Inbar et al., 2018). A series of pollen sequences from the Pleistocene and into early Holocene show that olive populations oscillated but were consistently present in the region (see Langgut et al., 2019 for details). For example, an extensive comparative study of deep core samples from the South Levantine Mediterranean Sea conducted by Langgut et al. (2011) showed a decrease in olive pollen during the last glacial period (75.5-56.3 kya BP), associated with a cold and dry climate, paralleled with minor pollen components from frost-sensitive Mediterranean evergreen species such as oak (*Quercus calliprinos* L.*)* (Miebach et al., 2017). An increase in olive pollen in the early Neolithic period, before the onset of agriculture, indicates an increase in olive trees likely due to warmer temperatures after the Younger Dryas (12,900 to 11,700 years BP) (Schiebel, 2013).

Changes in patterns of pollen demonstrate the impacts of anthropogenic activity on the vegetation structure of the region (Langgut et al., 2014; Schiebel and Litt, 2018). In the southeastern Mediterranean, the understanding of past vegetation and agricultural practice during the last 9,000 years was inferred from various palynological studies of sediments in Lake Kinneret (Baruch, 1986; Langgut et al., 2013; Schiebel, 2013; Langgut et al., 2015; Schiebel and Litt, 2018). The pollen pattern suggested that deciduous oaks (*Quercus* sp.) were replaced mainly by pines (*Pinus* sp.), evergreen oaks (presumably *Q. calliprinos*), and olive trees (Yasuda et al., 2000). This selective deforestation (9000-7000 BP) was arguably related to early settlement and anthropogenic demand for wood (Yasuda et al., 2000). Alternatively, Schiebel and Litt (2018) argued that the reduction in pollen of deciduous species during the Neolithic, as evident in the Dead Sea region (Litt et al., 2012) and Lake Kinneret (Schiebel and Litt, 2018), can be associated with increased aridity during the Holocene.

The onset of olive cultivation is reflected in a comprehensive comparison of pollen records from 23 locations around the Mediterranean (Israel, Lebanon, Turkey, Greece, Italy, Spain, and Portugal) published by Langgut et al. (2019). This study convincingly points to a southeastern Mediterranean origin of olive cultivation beginning around 6,500 years BP. Furthermore, the results of Langgut et al. (2019) support a general model suggesting that large-scale cultivation in the southeast Mediterranean preceded cultivation by approximately 3,000 years in the Northern Levant and the Aegean and Iberian peninsulas.

Pollen records indicate major changes in the southeastern Mediterranean vegetation landscape during the Early Bronze Age (~5900/5600-4500 BP) when the reduction in pollen of maquis trees (evergreen oak and pines) was paralleled with a massive increase in olive pollen (Baruch, 1986; Neumann et al., 2007a; Neumann et al., 2007b; Finkelstein and Langgut, 2018; Schiebel and Litt, 2018). It has been argued that the rise of olive in Mediterranean pollen records provide some indication that a wetter climate enabled the expansion of olive cultivation in the region (Langgut et al., 2016). Therefore, it can be assumed that cultivar selection, knowledge of horticulture, and vegetative propagation—prerequisites for this type of cultivation—were already in use by this period. In addition, the enormous concentrations of pollen during this period (Neumann et al., 2007a; Langgut et al., 2013; Langgut et al., 2014; Langgut et al., 2015; Langgut et al., 2016) indicate that olive production exceeded the amount needed by the local society, thus suggesting that the exploitation of secondary products (olive oil and possibly table olives), and presumably trade of olive products during this period (Langgut et al., 2016).

About a millennium later in the Late Bronze Age (~1500-1100 BCE), a decrease in arboreal pollen, including olive, and an increase in Poaceae pollen appears indicative of a drier climate and change in the agricultural landscape (Langgut et al., 2015). The climatic conditions through this period are described in the texts (best described in sources from the Northern Levant and Mesopotamia) as a time of drought and famine, which led to political instability and the destruction of cities, consequently decreasing the cultivation of olives (Langgut et al., 2015; Finkelstein et al., 2017). Subsequent pollen records from the Iron Age (c. 1100-583 BCE) showed an increase in Mediterranean vegetation throughout the period, indicating an improvement in climatic conditions (Langgut et al., 2015).

Overall, the pollen records and complementary archaeobotanical information (below), are strong evidence for the onset, development and spread of olive cultivation, its anthropogenic impact on local vegetation (Finkelstein and Langgut, 2018; Schiebel and Litt, 2018), and how it paralleled larger socio-political changes in the region (Langgut et al., 2015; Finkelstein et al., 2017; Langgut et al., 2019).

### 2.2 Archeological evidence

Evidence gleaned from archeological sites, olive oil extraction facilities, and archaeobotanical remains of olive pits and wood, as well as ancient literature (reviewed by Goor, 1966), can help us reconstruct the history of agriculture, including which crops were cultivated, understanding local farming communities, and the impact of horticulture on the local economy, among others (Kamlah and Riehl, 2020). The first archaeobotanical evidence for possible experiments in cultivation come from the Epipaleolithic site of Ohalo (c. 23000 BP), a submerged site in Lake Kinneret (Snir et al., 2015). Less controversially, the more accepted date for the onset of cereal cultivation in the eastern Mediterranean begins around 12000 BP (Zohary et al., 2012). However, it is possible that initial farming for small-scale food production occurring earlier was based on the deliberate planting and harvesting of CWR, mainly the precursors to barley and wheat (Edwards, 2020).

The initial cultivation of fruit trees, and olives, occur a few millennia later. Evidence for collecting olives is known from at least the Epipaleolithic, again from Ohalo, suggesting that these fruits were exploited as a staple food and a source of fat as early as 23000 BP (Weiss et al., 2004; Zohary et al., 2012).

Thousands of crushed olive pits found at the submerged Kfar Samir archeological site, on the Mediterranean coast, provides the earliest evidence (~7,600–7,000 BP) for olive oil production (Galili et al., 1997). Indeed, the presence of plant lipids in residue analysis of storage vessels from the same period excavated in the Galilee (Ein Zippori) supports the identification of the exploitation of olives for oil (Namdar et al., 2015). Not far from Kfar Samir, at the Hishuley Carmel site (6700–6500 BP), underwater surveys revealed the presence of large amounts of olive pits in elliptical stone structures, suggested by the authors to be the oldest and first indication of fruit preservation and pickling (Galili et al., 2021).

Olive pits of naturally growing trees, either var. *sylvestris* or feral trees of cultivated olives, have small fruits and higher variability in their dimensions than their domesticated descendants (Dighton et al., 2017; Galili et al., 2021; Terral et al., 2021). Olive pits at both Kfar Samir and Hishuley Carmel (above) show considerable variation in morphological features and resemble those of wild var. *sylvestris* populations, which today grow near these sites (cf. Barazani et al., 2016). This suggests that olive fruits during the Pottery Neolithic and early Chalcolithic sites were most probably gathered from naturally growing trees rather than cultivated (Kislev, 1995; Galili et al., 2021; Terral et al., 2021).

Traditionally, the strongest evidence for well-established cultivation was the evidence of olive pits and charred olive wood from Chalcolithic sites outside their natural distribution areas (Zohary and Spiegel-Roy, 1975; Liphschitz et al., 1996; Van Den Brink et al., 2001; Langgut and Garfinkel, 2022). This includes the famous site of Teleilat Ghassul in the Dead Sea region (Zohary and Spiegel-Roy, 1975), as well as others such as Tel Tsaf (Langgut and Garfinkel, 2022), Abu Hamid and Tell esh Shuna (Neef, 1990). However, since the Chalcolithic/Bronze Age transition was characterized by changes to technological and transportation (Milevski, 2013), the possibility of long-distance trading in olive products and local cannot be ruled out.

Looking at the morphological data from olive stone assemblages, those excavated at Teleilat Ghassul (upper Jordan Valley) do indeed show a decrease in morphological variability towards the end of the Late Chalcolithic (Meadows, 2005), indicative of cultivation around 6400 BP. In a similar study at Pella (southern Jordan Valley), a morphometric analysis of olive pits from the Pottery Neolithic to the Iron Age (6200–800 BCE) was used by Dighton et al. (2017) to investigate the long history of olive cultivation in the southern Levant. The results show a decrease in variation in pit length and width over time, with the authors suggesting that olive cultivation only begins at the site in the Early Bronze Age (Dighton et al., 2017). During the Early Bronze Age, the increase in the number of pressing facilities and oil-storing vessels indicated a rising importance of olive horticulture and its impact on human society, one very much influenced by demands of Old Kingdom Egypt and trade (Kamlah and Riehl, 2020). However, it is important to note that in contrast to the palynological records (above), archaeobotanical evidence for olives from the Bronze Age occurs at lower proportions to other important crops (i.e., wheat and barley). This contradiction can be mostly explained by the fact that wheat and barley are self-pollinating cereals, as well as the low taxonomic resolution of Poaceae pollen in general. As wind pollinators of self-incompatible species, the high dispersal of olive pollen also obscures the actual magnitude of olive cultivation.

Additionally, archaeological site formation processes – both natural and anthropogenic (cf. Schiffer, 1987; Shahack-Gross, 2017) – bias the presence and preservation of olive remains in the archaeological record. For example, anthropogenic activities such as olive oil processing and the utilization of waste products for fodder and fuel, remove olive seeds from the archaeological record. In general, with notable excavations at rare olive processing sites, archaeobotanical remains likely represent domestic consumption of table olives (Kamlah and Riehl, 2020).

The climatic conditions, paleoclimatic data (Bar-Matthews et al., 1998; Bar-Matthews et al., 1999; Bar-Matthews et al., 2003; Bar-Matthews and Ayalon, 2011), and the palynological studies (above) indicate that agriculture in the southeast Mediterranean flourished during the Bronze Ages. However, the Intermediate, Middle, and Late Bronze Ages, and especially the succeeding Iron Age, were characterized by fluctuating climatic conditions that resulted in periods of increased aridity (above). Thus, the agricultural practice during these periods relied on crops with high tolerance to water deprivation and salinity (Kamlah and Riehl, 2020), which probably required a selection process of the main annual crops and olives, choosing specimens well-adapted to local stress conditions.

## 3 Naturally growing olive trees in the southeast Mediterranean: Genetic evidence for the existence of a wild olive ancestor

**Figure 1:**
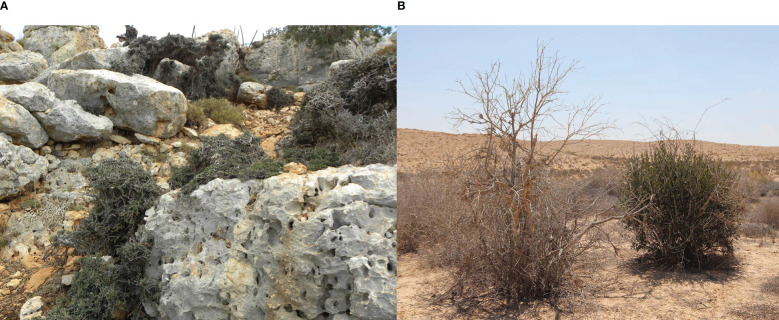
Naturally growing olives trees in the Galilee, Israel **(A)** showing a bushy Bonsai form with juvenile leaves, **(B)** a feral individual growing in the Negev desert.

Naturally growing olive trees show remarkable phenotypic differences from cultivated varieties, possessing significantly smaller fruits (presumably with lower oil content), high variation in fruit morphology (e.g., Galili et al., 2021; Terral et al., 2021), and possessing a bushy nature often with a long juvenile stage (**Figure 1A**). However, the long co-existence of cultivated olive trees alongside their wild relatives, which provided opportunities for hybridization between the two, problematizes whether existing wild populations represent genuine examples of var. *sylvestris*, or are merely feral populations. Consequently, the genetic structure of modern populations of non-cultivated olive trees can be strongly influenced by cultivation in adjacent olive groves (Barazani et al., 2016).

Using molecular markers, several studies investigated the genetic relationships between cultivated olives and the supposedly wild var. *sylvestris* in an attempt to better understand the olive tree domestication process (Baldoni et al., 2006; Kaniewski et al., 2012; Besnard et al., 2013b; Diez et al., 2015). In addition, new generation sequencing and molecular marker techniques offer novel means to identify populations of var. *sylvestris* trees and differentiate between wild and feral populations (Besnard et al., 2001; Breton et al., 2006; Besnard et al., 2013a; Besnard et al., 2013b; Diez et al., 2015; Unver et al., 2017; Gros-Balthazard et al., 2019). Moreover, the assembled genomes of var. *sylvestris* (Unver et al., 2017) and old cultivated trees of the Farga cultivar (Cruz et al., 2016), as well as several transcriptome studies (Munoz-Mérida et al., 2013; Carmona et al., 2015; Iaria et al., 2016; Sarah et al., 2017), and genotyping by single nucleotide polymorphism (SNPs) (Mariotti et al., 2020) have been recently used as the basis for a wide transcriptome screen of var. *sylvestris* and cultivated accessions, revealing the genomic consequences of olive domestication (Unver et al., 2017).

**Figure 2:**
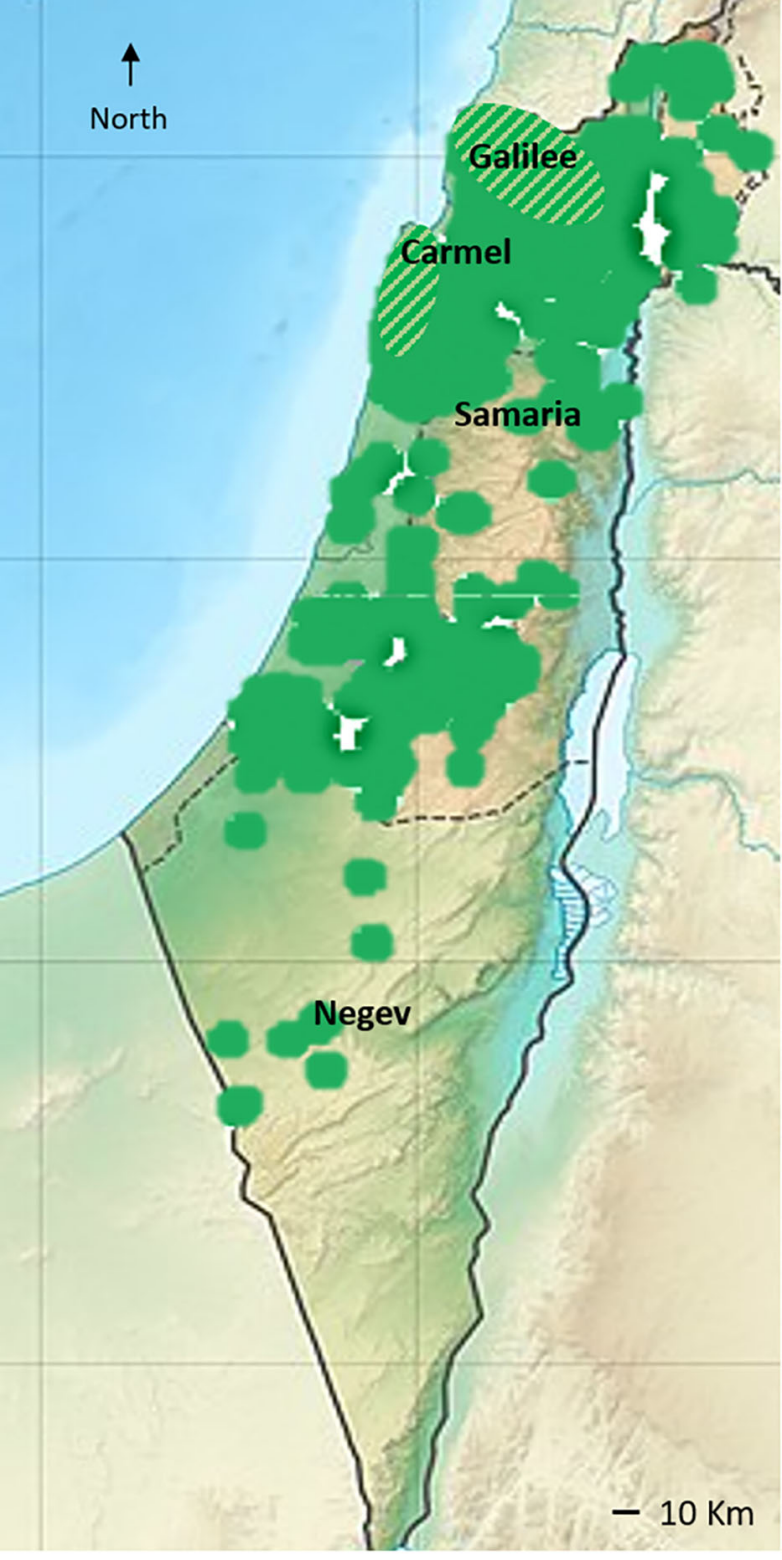
The distribution of cultivated olive landraces and naturally growing trees in the area of Israel and the Palestinian Authority (presented in green color). Information was gathered from the BioGis, Israel biodiversity website (https://biogis.huji.ac.il/eng/home.html). Naturally growing feral trees are found throughout the cultivation area (Barazani et al., 2016), while genetic evidence of populations of the wild var. *sylvestris* were found in the presumed hypothetical distribution range of wild olives (Zohary et al., 2012) (diagonal stripe lines).

In the southeast Mediterranean, the hypothetical southern distribution of the wild var. *sylvestris* includes the Galilee and the Carmel mountain range (Zohary and Spiegel-Roy, 1975; Zohary et al., 2012). More recently, a species distribution model using temperature and the locations of wild-growing olive groves as defining variables was used by Besnard et al. (2013b) to infer the present distribution of wild var. *sylvestris*. Notably, the model discarded locations of nearby cultivated groves, thus excluding the possibility of feral populations. The resulting map (Besnard et al., 2013b) inferred suitable habitats in the Galilee, Carmel, Samaria, and Judean mountain ranges. However, as these geographical regions are typical of traditional olive cultivation (see Barazani et al., 2014; Barazani et al., 2017), the resulting map differs from the hypothetical distribution range of wild olives in the southeast Mediterranean suggested by Zohary et al. (2012) (**Figure 2**).

In a previous study, using genetic diversity parameters derived from 15 SSR (simple sequence repeats markers, microsatellites), Bayesian clustering with the admixture model, and Rousset’s genetic clustering, we differentiated genetically between feral, neglected cultivated olive trees in abandoned groves and populations of what look like wild var. *sylvestris* (Barazani et al., 2016). In support of the distribution range of wild olives in the region suggested by Zohary et al. (2012), naturally growing trees sampled in the Judean mountains were identified as feral trees (Barazani et al., 2016), as well as in the Negev desert (**Figure 1B**). However, populations from the Galilee and Carmel showed distinct genetic differentiation from feral trees and the main cultivated landraces (below), indicating that wild populations of var. *sylvestris* still exist in these regions (**Figures 1**, **2**).

## 4 Olive landraces of the southern Levant

Traditional olive groves in the southern Levant are mainly concentrated in the coastal, inland, and mountainous areas of the southern Mediterranean districts of the region (i.e., modern Lebanon, Israel, the Palestinian Authority, and Jordan), areas with more than 350 mm of rainfall/year (see the distribution map of olives in Israel in **Figure 2**). However, olive exploitation since the Bronze Age (above) expanded the cultivation zone of olives to more semi-arid and arid zones of the eastern Mediterranean (<300 mm rainfall/year), as exhibited by ancient living olive trees in the Negev desert, relicts of Byzantine agricultural system (Tepper et al., 2022) (**Figure 2**). Thus, the range of environmental conditions in the region (Goldreich, 2003) suggests that human activity throughout the long history of olive cultivation may have selected genotypes exhibiting adaptive tolerance to climatic stress conditions, especially water stress (Barzilai et al., 2021). In the era of drastic global climate change, such clones/cultivars could determine the future of olive production.

Estimations for the number of olive cultivars vary widely, from several hundred (Olea database, http://www.oleadb.it/) to around 2,600 (Rugini and Lavee, 1992). Olive cultivars are distinguished by several morphological characteristics of the fruits and leaves, tree shape, oil characteristics, and their utilization (e.g., use for oil or table olives). In recent years, the use of molecular marker techniques, especially SSRs and SNPs, proved to be reliable for cultivar differentiation and identification (Rallo et al., 2000; Sefc et al., 2000; Cipriani et al., 2002; Essadki et al., 2006; Besnard et al., 2011). Thus, the employment of molecular marker techniques proved efficient in characterizing olive germplasms (Khadari et al., 2003; Loumou and Giourga, 2003; Belaj et al., 2003a; Belaj et al., 2003b; La Mantia et al., 2005). These studies included molecular identification of traditional local southeast Mediterranean cultivars while also revealing the genetic relationships among cultivars originating from around the Mediterranean Basin (Owen et al., 2005; Haouane et al., 2011; Díez et al., 2016; and others).

Using a multi-locus lineage (MLL) analysis of 15 SSR markers, we previously reported on a genetic survey of ancient living olive trees in Israel and the Palestinian Authority (Barazani et al., 2014). All trees included in the survey were selected to represent old trees (assumed by trunk perimeter >2 m) growing in traditional olive groves. The MLL analysis considered a mutational threshold, thus excluding genetic differences due to somatic mutations, which enabled the identification of clonal identity. The findings indicated that the majority of living old olive trees (90%) in the southeast Mediterranean region belong to a single MLL (MLL1), one associated with the most common and widely dispersed east Mediterranean cultivar, Souri (Barazani et al., 2014). The Souri cultivar, typical to the southern Levant, occupies most of the traditional rain-fed olive groves. Thus, it is highly adaptable to varying climatic and semi-arid conditions, shallow and stony marginal soils (Ben-Ari et al., 2014), and occasional droughts.

Twenty-seven other local olive cultivars were described in the first half of the 20^th^ century by Goor (1948). These are divided in current terminology into five main cultivars: Nabali Baladi, Nabali Muhassan, Mailsi, Souri, and Souri Rumi (Barazani et al., 2008). Local olive growers consider the latter cultivar to be a remnant of olive trees from the Roman period. Supporting the existence of specific unique clones/cultivars, the MLL analysis identified the existence of several other clonal groups among ancient living trees, as well as a second large MLL group (MLL7) that was pronounced as a rootstock of old grafted trees (Barazani et al., 2014). Testing the potential contribution of MLL7 as a rootstock, a linear regression analysis revealed that the spread of MLL7 in the southeast Mediterranean decreases with increasing aridity (Barazani et al., 2017). Thus, the distribution of MLL1 grafted onto MLL7_rootstock_ in old olive trees was more pronounced in high elevations mesic regions (Galilee and Carmel), whereas the distribution of the Souri cultivar (MLL1) as non-grafted old trees increased in regions outside the natural Mediterranean olive cultivation zone. In previous studies (Tugendhaft et al., 2016; Barzilai et al., 2021), we tested MLL1’s response to drought compared to Barnea cultivar trees of the same age and the Spanish drought-resistant Picual cultivar (Shaheen et al., 2011). Monitoring several physiological traits including stomatal conductance, net photosynthesis, leaf water potential and stem growth (Tugendhaft et al., 2016) and soil volumetric water content, stem water potential, and gas exchange (Barzilai et al., 2021), the results of the two studies pointed to the higher drought tolerance of the Souri cultivar. Thus, the selection of the Souri cultivar supports the hypothesis that the selection of the Souri cultivar enabled the expansion of the olive cultivation zone in the southeast Mediterranean into more arid habitats.

**Figure 3:**
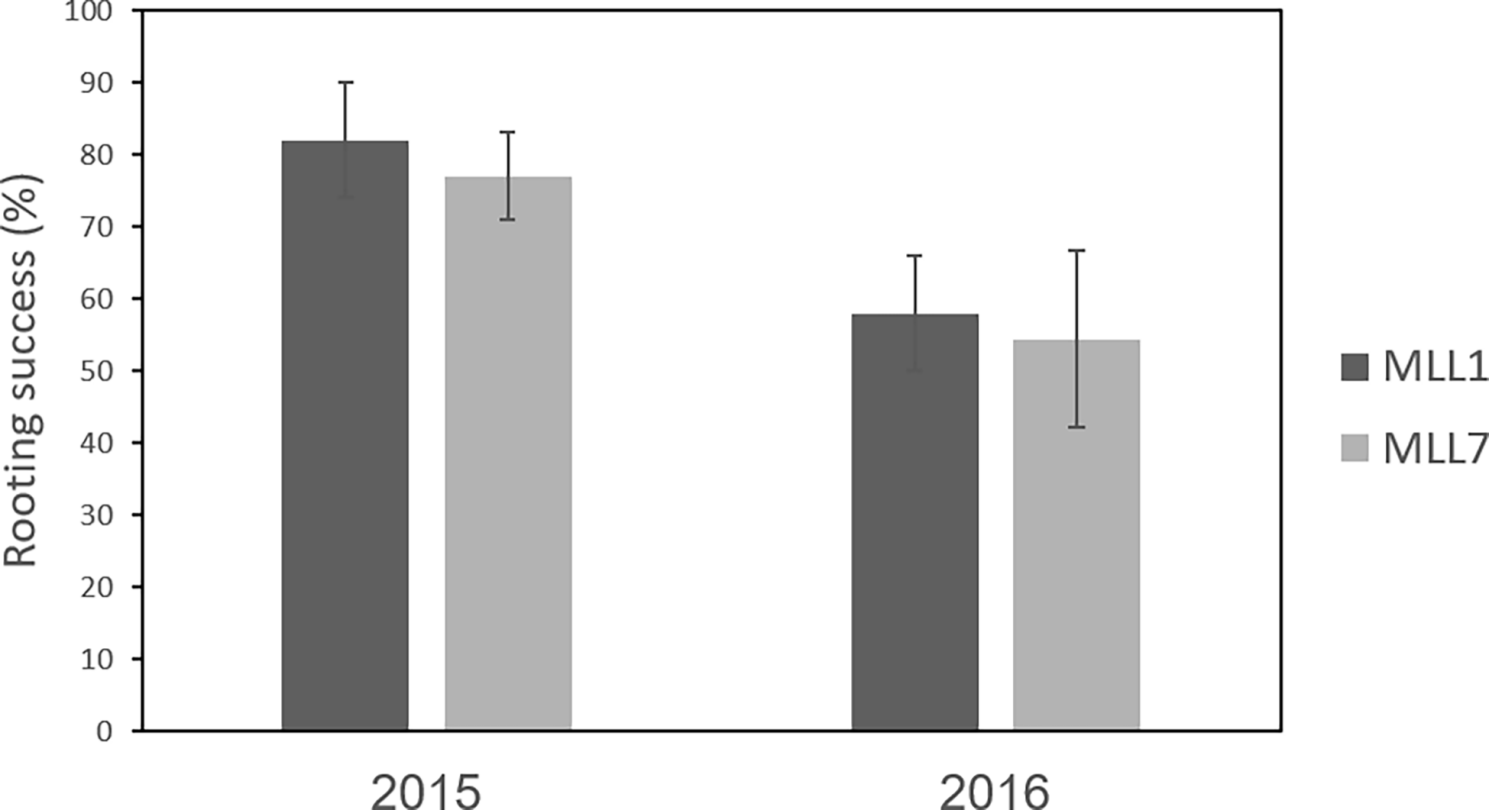
Rooting success (average ± std) of cuttings of MLL1 and MLL7. Leaf cuttings were taken from trees growing in a live germplasm collection (Gilat Research Center, Israel) and rooting success was evaluated over two subsequent years.

The grafting of olives is thought to increase the survival of the propagated trees (Foxhall, 2007), thus improving the propagation success of cultivars that do not root easily, such as the south Levantine Souri (MLL1) cultivar. However, the rooting capability of MLL1 was not significantly different in comparison to MLL7 (**Figure 3**), suggesting that MLL7 does not facilitate propagation. The use of certain wild-growing olive trees as a source of rootstock that increases tree vigor was previously reported in Turkey (Zohary et al., 2012). Rootstock genotype has been shown to influence olive growth and tolerance to Fe deficiency in calcareous soils (Alcantara et al., 2003). In addition, the results of an Akaike information criterion (AIC) model selection procedure also indicate that grafting of the common Souri cultivar (MLL1) on MLL7_rootstock_ positively improved oil quality under certain environmental conditions (Barazani et al., 2017). However, whether grafting increased tree vigor in the southern Levant, or provided an easy means for propagation, remains unclear.

**Figure 4:**
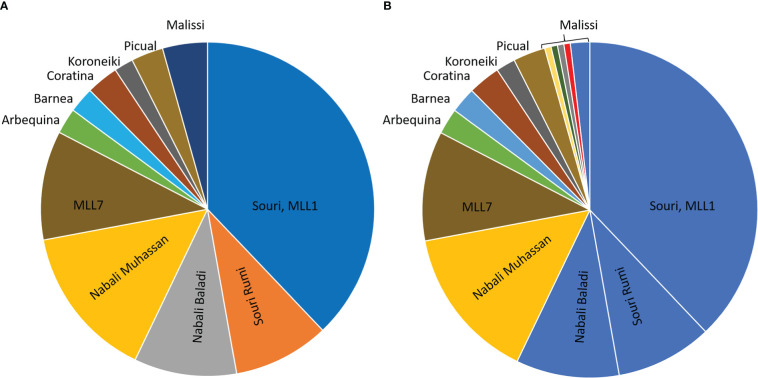
Results of the Multi-locus lineage (MLL) analysis of olive trees growing in traditional groves. **(A)** The analysis included trees that were sorted by their cultivar names: Souri (n=61), Souri Rumi (n=15), Nabali Baladi (n=16), Nabali Muhasan (n=24), and Malisi (n=7). The analysis also included representative samples belonging to MLL7 genetic group (n=17) (Barazani et al., 2014), and several Mediterranean cultivars, Arbequina, Coratina, Koroneiki, Picual, and Barnea (n=3-5). **(B)** The MLL characterization in the pie diagram is presented according to colors, e.g. Souri, Souri Rumi, Nabali Baladi and Malisi (n=4) were clustered together in the same MLL1 genetic group.

As an additional line of investigation, we report here on the genetic identity of 124 olive trees growing in traditional groves in the southern Mediterranean. The clonal identity of the trees was tested in reference to their cultivar name, identified by the growers as Souri, Souri Rumi, Nabali Baladi, Nabali Muhassan, and Mailsi (**Figure 4A**). The results of genetic clonal identification (MLL analysis) revealed that all trees that the growers identified as Souri, Souri Rumi and Nabali Baladi, as well as three trees that the growers identified as Malisi, were clustered together in the MLL1 genetic group (**Figure 4B**), thus supporting synonymous terminology. A second sizeable genetic group included trees belonging to the Nabali Muhassan cultivar (**Figure 4B**). It is important to note that during sampling, the growers and owners of the olive groves pointed to trees that were propagated from the same cultivar but possess different phenotypic traits and thus were named differently. In addition, four trees identified by the growers as being Malisi were separated into single occurrence MLLs, different from the main three local genetic groups, i.e., MLL1 namely Souri, MLL7, and Nabali Mohassan, as well as the reference cultivars Barnea, Arbequina, Coratina, Koroneiki and Picual (**Figure 4B**). Thus, the genetic survey of the local olive germplasm (**Figure 4**; Barazani et al., 2014) identified unknown clones/cultivars with presumed agronomical potential that has yet to be studied.

## 5 Summary and new perspectives

Overall, the tangible evidence summarized above indicates that the earliest indication of olive cultivation was found in the southern Levant. As this part of western Eurasia is generally considered one of the domestication hotspots for many crops (e.g., emmer wheat, barley, garlic, lettuce, among others) (Zohary et al., 2012), studies that aimed to decipher olive domestication, (e.g., Lumaret et al., 2004; Breton et al., 2008; Besnard et al., 2013b; Diez et al., 2015) included local cultivars, and several naturally growing olive populations from the southeast Mediterranean. These included local landraces (Souri and Nabali) but, in some cases, also modern local southeast Mediterranean varieties such as Barnea, Merhavia, and Ma’alot (see Besnard et al., 2013a and Diez et al., 2015). In addition, we previously showed that adjacent olive cultivation could strongly influence the genetic structure of modern populations of non-cultivated olive trees (Barazani et al., 2016). Thus, such studies may have wrongly used biological material from feral populations. We suggest that our genetic survey of naturally growing olive populations (Barazani et al., 2016), the unique local olive clones identified by our molecular approach (above), may represent previously unknown ancient genetic stock of early cultivated olives and thus might be utilized to add to the debates over olive domestication. More importantly, underutilized clones selected to withstand harsh local conditions can be reintroduced as better-adapted crops fitted to the changing environment or used as a source for desirable new traits in breeding programs.

## Author contributions

OB and ZD wrote the manuscript with contribution from AD. All authors edited and approved the final manuscript.
